# Faecal Immunochemical Test (FIT) Value-Based Algorithm to Triage Symptomatic Colorectal Patients: A Retrospective Study From a Tertiary Care Hospital

**DOI:** 10.7759/cureus.69889

**Published:** 2024-09-21

**Authors:** Amal Boulbadaoui, Ayesha Bibi, Guleed Mohamed, Anne Gaunt, Philip Varghese, Muhammad Umair Rashid

**Affiliations:** 1 Colorectal Surgery, University Hospitals of North Midlands National Health Service Trust, Stoke-on-Trent, GBR; 2 General Surgery, University Hospitals Birmingham National Health Service Trust, Birmingham, GBR

**Keywords:** colorectal cancer, diagnostic test accuracy, faecal immunochemical testing, quantitative fit test, colonoscopy

## Abstract

Background

The aim was to evaluate the diagnostic accuracy of quantitative faecal immunochemical testing (FIT) in diagnosing colorectal cancer in symptomatic patients and using it to prioritize patients for urgent colorectal investigations.

Methods

A retrospective review was done of all symptomatic, FIT-positive patients referred from primary care to the colorectal clinic as per the National Institute for Health and Care Excellence (NICE) DG30 pathway from November 1, 2021, to February 11, 2022. Patients under 18 years of age were excluded. All patients were triaged to test (TTT) or booked in face-to-face (F2F) clinics according to a local algorithm. The data was collected in Microsoft Excel (Microsoft Corporation, Redmond, Washington, United States), and statistical analysis was performed using IBM SPSS Statistics for Windows (IBM Corp., Armonk, New York, United States).

Results

Out of 915 FIT-positive patients, 774 (84.5%) patients were TTT and 70 (7.65%) were booked in F2F clinics. The mean age was 71 years, and the majority were females (n=488 (F:M = 53:47)). However, a higher prevalence of positive FIT test was observed in men than in women at higher cut-off values of >100 and >200 μg Hb/g faeces (18.79 % vs 17.48% and 12.78% vs 11.69%, respectively). The number needed to scope was 10.8, 6.03, and 5.20 at cut-off values of ≥10, >100, and >200 μg Hb/g, respectively. At cut-off values of ≥10, >100, and >200 μg Hb/g, the specificity for colorectal cancer (CRC) with a 95% confidence interval (95% CI) was 63.75 %, 88.10%, and 92.27 %, respectively, and the positive predictive value (PPV) was 9.63, 18.20, and 21.24, respectively. The majority of the patients (30.9%) had no pathology on colonoscopy, whereas CRC was detected in 9.8%.

Conclusion

Quantitative FIT in symptomatic colorectal patients can be used to triage patients to investigations more appropriately, reducing the burden on outpatient and endoscopy units and improving the overall efficacy of health provision.

## Introduction

Colorectal cancer (CRC) remains a significant global health concern with approximately 42,900 new cases every year in the United Kingdom (UK) alone. It is the second most common cause of cancer deaths [1]. Early detection is crucial for positive patient outcomes as the survival rate for stage 1 CRC is >90% compared to <10% in stage 4 [2].

While colonoscopy remains the gold standard for CRC detection, the increasing number of referrals poses a great challenge to already limited colonoscopy unit resources. Moreover, 60-80% of symptomatic patients do not have CRC and are unnecessarily exposed to an invasive procedure and complication risks [3,4] with high costs [5].

Faecal immunochemical testing (FIT) was introduced in 2017 by the National Institute for Health and Care Excellence (NICE) (diagnostic guidance 30 (DG30)) [6] as a cost-effective, non-invasive method to triage CRC referrals in primary care for symptomatic patients [7]. FIT detects occult blood in stool. Notably, the NICE guidelines recommend using a threshold above 10 μg Hb/g for low-risk symptomatic patients [6]. However, ongoing debates surround this recommendation as FIT has proved to miss early stage 1 cancer and precancerous polyps that could be easily removed in colonoscopy, eliminating the need for surgery or chemotherapy [8].

**Figure 1:**
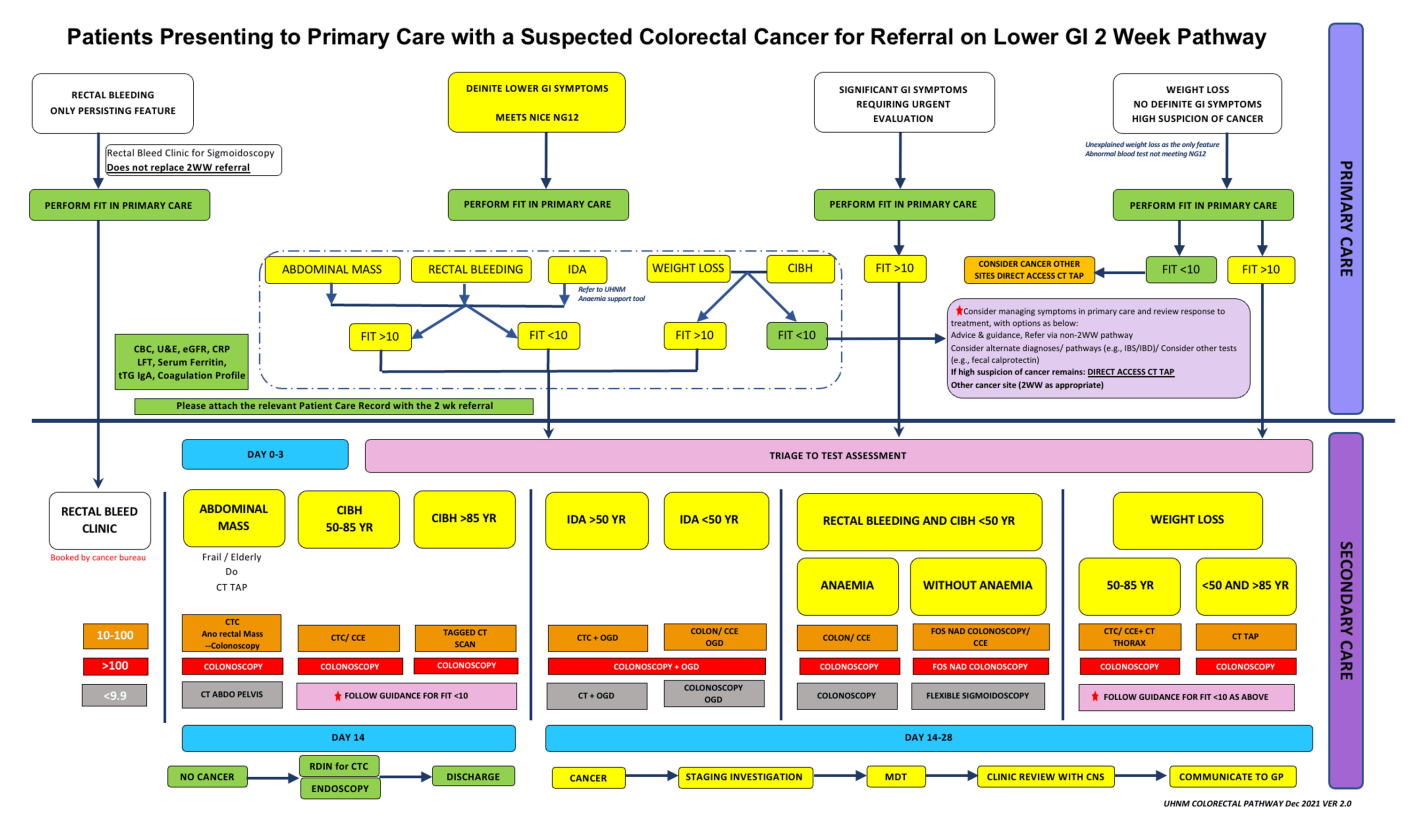
Triage-to-test pathway at University Hospitals of North Midlands National Health Service (NHS) Trust, North Midlands, United Kingdom 2WW, two-week wait; FIT, fecal immunochemical test; IDA, iron deficiency anemia; CIBH, changes in bowel habits; CT TAP, CT thorax, abdomen, and pelvis; FBC, full blood count; U&E, urea and electrolytes; eGFR, estimated glomerular filtration rate; LFT, liver function test; tTG IgA, tissue transglutaminase IgA; IBS, inflammatory bowel syndrome; IBD, inflammatory bowel disease; CTC, CT colonography; CCE, colon capsule endoscopy; OGD, oesophagogastroduodenoscopy; FOS, flexible sigmoidoscopy; NAD, no active disease; CT ABD, CT abdomen; MDT, multidisciplinary team, CNS, clinical nurse specialist; GP, general practitioner Image Credit: Author Philip Varghese

The FIT result is a mandatory part of referral based on the DG30 guidelines in University Hospitals of North Midlands National Health Service (NHS) Trust, North Midlands, UK. The referrals are then triaged to appropriate tests based on a locally designed algorithm (Figure 1). The algorithm caters to different values of FIT results and different symptoms according to the DG30 guidelines. The patients are triaged based on both categories for appropriate investigations. 

This study aimed to comprehensively evaluate the diagnostic accuracy of FIT in identifying colorectal cancer at different cutoffs along with the frequency of other bowel pathologies including adenoma, diverticular disease, and inflammatory bowel disease (IBD). Secondly, we wanted to validate the algorithm used to triage patients straight to test saving face-to-face (F2F) clinic appointments.

## Materials and methods

The study was done at Royal Stoke University Hospital, University Hospitals of North Midlands NHS Trust, Stoke-on-Trent, UK. It was a retrospective review of all symptomatic, FIT-positive patients over 18 years of age who were referred as per the NICE DG30 pathway to the colorectal clinic from primary care between November 1, 2021, and February 11, 2022. FIT positivity was set at ≥10 μg Hb/g of faeces, as per the NICE DG30 guidelines [6]. Patients were investigated and risk-stratified in accordance with their FIT result and presenting symptoms as per a local algorithm (Figure 1).

A total of 915 FIT-positive patients were triaged between these dates, and all were included in the study. Each patient was then triaged to test (TTT) or booked in F2F clinics according to the algorithm (Figure 1). The triage process was carried out in dedicated triage clinics facilitated by colorectal consultants, specialist nurses, and speciality doctors. The data was collected on Microsoft Excel (Microsoft Corporation, Redmond, Washington, United States), and patients were divided into groups based on their level of FIT results: ≥10, >100, >200, and 100-200 μg Hb/g. The ≥10 μg Hb/g group had all the patients in the study, whereas the >200 μg Hb/g group only had patients with FIT values >200 μg Hb/g. Statistical analysis was undertaken using IBM SPSS Statistics for Windows (IBM Corp., Armonk, New York, United States) to determine the sensitivity and specificity.

## Results

Out of 915 patients, 774 (84.5%) were TTT, while 70 (7.65%) were booked in F2F clinics. Table 1 illustrates the demographics of patients in various FIT subgroups. 

**Table 1:** Basic demographics of different subgroups FIT, faecal immunochemical testing; M:F, male:female; F2F, face to face

	Number of patients	Median age (years)	Gender (M:F)	Type of clinic (% of patients triaged in F2F clinics)	% of patients triaged by consultants
≥10	915	71	427:488	7.6	44.6
>100	332	71	172:160	10.5	46.6
>200	224	70	117:107	9.8	47.7
100 to 200	108	73	55:53	12	44.4

The mean age of patients was 71 years, consistent across all subgroups. Among the 915 FIT-positive patients, 488 (53.33%) were women. There was a positive correlation observed between increasing age and FIT positivity; 129 out of 396 patients (32.58%) under the age of 50 years tested positive, compared to 206 out of 402 patients (51.24%) over the age of 80 years (p <0.001). Higher cut-off values of >100 and >200 revealed a higher prevalence of positive FIT tests among men than in women, with 172 vs 160 patients (18.79% vs 17.48%) and 117 vs 107 patients (12.78% vs 11.69%), respectively. The least number of patients were seen in the FIT value range of 100-200 μg Hb/g faeces. Additionally, 408 patients (44.6%) were triaged by the consultants, while the remaining patients were triaged by speciality doctors and specialist nurses.

Table 2 outlines the frequency of bowel pathology in FIT-positive patients undergoing colonoscopy.

**Table 2:** Frequency of bowel pathology confirmed on colonoscopy

Diagnosis	N	%
Normal	283	30.9
Adenoma	204	22.2
Diverticular disease	139	15.1
Inflammatory bowel disease	25	2.7
Colorectal cancer	89	9.8
Others (haemorrhoids, colitis, proctitis, angiodysplasia, etc.)	104	11.3

The majority of patients, 283 (30.9%), had no pathology on colonoscopy. Among the positive findings, CRC was detected in 89 patients (9.8%), while adenomatous polyps were detected in 204 patients (22.2%). Other benign pathologies included diverticular disease in 139 patients (15.1%) and IBD in 25 patients (2.7%). Additionally, 104 patients (11.3%) had colonoscopy findings, which were grouped together as miscellaneous benign bowel diseases including haemorrhoids, colitis, proctitis, angiodysplasia, etc.

The diagnostic accuracy of FIT for CRC at different cut-offs of >10, >100 and >200 is summarised in Table 3. At these cut-offs, the specificity and positive predictive value (PPV) for CRC with 95% confidence intervals (95% CIs) increased for higher cut-off values. The number of patients required to undergo colonoscopy to detect 1 CRC also known as the number needed to scope was inversely proportional to the FIT cut-off values of ≥10, >100 and >200 μg Hb/g; this was 10.8, 6.03 and 5.20, respectively.

**Table 3:** Diagnostic accuracy of quantitative faecal immunochemical testing (FIT) at various cut-off values CI, confidence interval; PPV, positive predictive value; NPV, negative predictive value; TP, true positive; FN, false negative; FP, false positive; TN, true negative

	Sensitivity % (95% CI)	Specificity % (95% CI)	PPV %	NPV %	Positive likelihood ratio	Number needed to scope (NNS)	TP	FN	FP	TN	
≥10	92.7 (85.5 - 97)	63.74 (61.7 – 65.7)	9.63	99.53	2.56	10.8	89	7	826	1452
>100	63.5 (53.09 - 73.13)	88.10 (86.7 - 89.4)	18.20	98.31	5.34	6.03	61	35	271	2007
>200	50.0 (39.62 - 60.38)	92.27 (91.1 - 93.3)	21.24	97.79	6.47	5.20	48	48	176	2102
100 to 200	13.5 (7.41 -22.04)	95.8 (94.9 - 96.6)	11.92	96.38	3.25	11.94	13	83	95	2183

The adenoma detection rate was not significantly different statistically among these groups. For patients with FIT ≥10 μg Hb/g, adenomas were detected in 204 out of 915 patients (22.2%), compared to 55 out of 224 patients (24.5%) with a FIT level >200 μg Hb/g.

## Discussion

NHS England outlined its latest faster diagnostic standards (FDS) framework which includes the use of FIT in primary care as set out in guidelines by the Association of Coloproctology of Great Britain and Ireland (ACPGBI) [9]. We compared different cut-off values for FIT in symptomatic patients with the aim to more appropriately tailor investigations according to various symptoms and presentations and prioritise patients for urgent colorectal investigations.

The median age of FIT-positive patients was 71 years which was higher than the median age of FIT-negative patients (66 years) in the same time period, a trend consistent with available literature [10]. Older age is an independent risk factor for a higher FIT result along with neoplasia itself [10].

As evidenced by the results, there is a notable rise in the prevalence of CRC with increasing faecal haemoglobin level, with a positive predictive value (PPV) of 22.4 % at a cut-off of >200 μg Hb/g compared to 9.63% at >10 μg Hb/g. This knowledge can help prioritise patients for colonoscopies, especially at times when resources are limited, such as was encountered during the COVID-19 pandemic. However, using a stringent cut-off value of FIT alone comes at the cost of missing CRC with sensitivity decreasing significantly from 92.7% to 50% at cutoff values of 10 μg Hb/g and >200 μg Hb/g, respectively. In contrast, patients referred based on symptoms alone for colonoscopy can be non-specific with the majority undergoing colonoscopy with no pathology identified. This can increase the burden on colonoscopy units as well as on outpatient clinics, further increasing the wait times.

A triaging pathway that integrates patients’ symptoms and FIT test results to optimise efficacy and resource utilisation is a useful tool in over-burdened healthcare systems. Our TTT algorithm, introduced during the COVID-19 pandemic, addresses this by triaging patients referred on CRC pathways (NICE DG30) based on symptoms and quantitative FIT results at various cut-off values. All symptomatic patients with a FIT result of >100 μg Hb/g were offered colonoscopy. For those with FIT results < 100 μg Hb/g or a negative FIT, triage to the most appropriate investigation was guided by their symptoms as outlined in NICE DG30 [6].

Anaemia is a well-recognised risk factor for CRC in primary care [11-13]. The relevance was further emphasised in a recent study [14] where four out of five patients diagnosed with CRC and FIT results <10 μg Hb/g presented with anaemia, either with or without iron deficiency. Consistently, a study in Dundee, Scotland, United Kingdom, corroborated similar findings with all patients found to have missed CRC with negative FIT (<10 μg Hb/g) found to have iron deficiency anaemia (IDA) [15]. In our TTT algorithm, patients <50 years old referred with IDA were directed to colonoscopy or computed tomography colonography (CTC) with oesophagogastroduodenoscopy (OGD) for FIT results ranging between 10 and 100 μg Hb/g. In contrast, patients aged >50 years were offered CTC and OGD across both FIT ranges (10-100 μg Hb/g and <10 μg Hb/g).

Change in bowel habits (CIBH) is one of the most common symptoms triggering referral, with 36 out of 89 CRC patients presenting with CIBH in isolation or with other abdominal symptoms. Despite this, several studies have suggested that CIBH alone lacks substantial association with CRC [16] and has a low PPV [17-20]. In contrast to anaemia, FIT emerges as a valuable tool in stratifying patients referred with CIBH, guiding the most appropriate investigation. A negative FIT can allow the exclusion of patients with CIBH over 50 years from the cancer pathway, facilitating their management in primary care or on a routine referral pathway. In our algorithm, patients presenting with CIBH were triaged to colonoscopy or CTC in 50-to-85-year-old patients. Tagged CT scans were suggested for patients >85 years old with a FIT value of 10-100 μg Hb/g to avoid side effects of full bowel preparation.

This study included a large sample size, providing robust data and increasing the statistical power. However, there are some limitations to this study. The retrospective cohort design limits the ability to adjust for confounding factors at the time of data collection. Additionally, the study relies on existing information, which may be incomplete or subject to inaccuracies, potentially leading to information bias. The population sample may not fully represent the general population, limiting the ability to generalise the results. Finally, the lack of long-term follow-up restricts our ability to evaluate the effectiveness of FIT over time.

## Conclusions

Quantitative FIT in symptomatic colorectal patients enables a more efficient allocation of diagnostic investigations and optimises the utilisation of healthcare resources. By implementing an algorithm-based triage system, patients can be directed to the most appropriate investigations based on their FIT results and presenting symptoms, which significantly reduces the rate of unnecessary invasive procedures like colonoscopy. The colonoscopy burden can be reduced by utilising alternative investigations like CTC in lower-risk patients. Moreover, the triage algorithm should be specifically tailored to incorporate the quantitative values of FIT results, ensuring that the stratification process accurately reflects the risk level associated with different FIT thresholds. This customization will further streamline the triage process, leading to more effective and timely patient management while optimising the overall efficiency of the healthcare system.
